# PARALLEL DEVELOPMENT OF MEMORY, DISABILITY, AND COMORBIDITY IN U.S. ADULTS AGE 65 AND OLDER

**DOI:** 10.1093/geroni/igz038.2442

**Published:** 2019-11-08

**Authors:** Nicholas J Bishop, Kaipeng Wang

**Affiliations:** 1 Texas State University San Marcos, San Marcos, Texas, United States; 2 Texas State University, San Marcos, Texas, United States

## Abstract

Cognitive health, physical function, and chronic disease represent interdependent health outcomes that may exert influence on the course of each other’s development. To investigate the association between baseline health in each domain and developmental change across domains, we estimated trajectories of working memory, mobility limitations, and comorbidity among US adults age 65 and older over 18 years. We drew observations from the nationally-representative Health and Retirement Study with an analytic sample consisting of 5,963 adults age 65 and over in 1998. Immediate word recall, an 11-item Nagi scale of mobility limitations, and a summary count of eight doctor-diagnosed chronic conditions were measured biennially from 1998 to 2016. Parallel-process quadratic growth models with individually-varying time scores were used to estimate non-linear trajectories of each health measure, allowing identification of associations between baseline health and developmental change in each health process at both earlier and later stages of older adulthood. All estimates adjusted for covariates, complex survey design, and missing data. Greater baseline immediate word recall was associated with less rapid increase in mobility limitations at earlier ages. More baseline mobility limitations were associated with faster increase in comorbidity at earlier ages. Greater baseline chronic conditions were associated with more rapid increase in mobility limitations at later ages. These results highlight the importance of conceptualizing health among older adults as an interdependent and developmental process and should help clinicians recognize that single-domain health status may influence the progression of other health outcomes at different stages of older adulthood.

